# Supplementary material for: Chemical (Alkali) Burn-Induced Neurotrophic Keratitis Model in New Zealand Rabbit Investigated Using Medical Clinical Readouts and In Vivo Confocal Microscopy (IVCM)
Source: Cells. 2024 Feb 22;13(5):379. doi: 10.3390/cells13050379 (PMC10931039; doi:10.3390/cells13050379)
Supplement: Supplementary file 1 [file cells-13-00379-s001.zip › cells-2677210-supplementary.pdf]

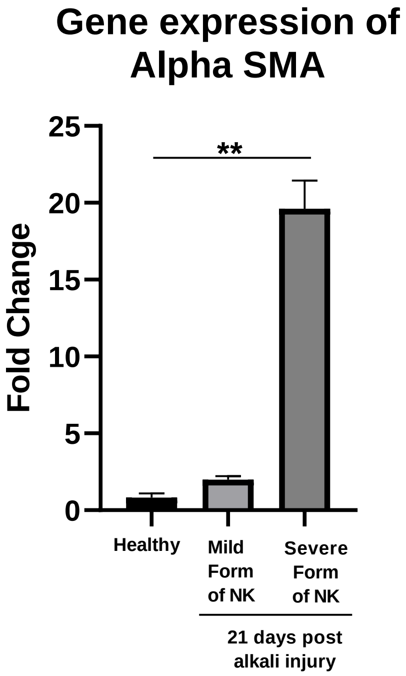

Figure S1. Real-time PCR shows the expression of alpha-SMA in the mild form and severe form of NK. Significant upregulation of the expression of alpha-SMA was observed in the severe form of NK as compared to the control and mild form of NK

Supplementary Table 1:

Primer Sequence for gene expression profiling using real time PCR (26)

Gene Name	Forward Primer	Reverse Primer
**GAPDH**	TGACGACATCAAGAAGGTGGTG	GAAGGTGGAGGAGTGGGTGTC
**Alpha SMA**	CTGACCGTATGCAGAAGGAAA	AGAAACAGAGCAGGGAAGTGAC

Supplementary Table 2:

Scheme of conversion from original machine readout to relative scoring

	Absolute values obtained from the instrument					
	Corneal Thickness (In µm) (0-5mm)	Transparency (In Gray Scale Unite) (0-5mm)	Epithelial Cell density (cells per mm^2)	Nerve Density (fibres per frame)	Keratocyte Density (cells per mm^2)	Nerve Sensation (In mm)
Healthy	348.9	20	4773	6	362	60
Mild Form of NK	347.5	35	4536	2.25	321	57
Severe Form of NK	694.5	82	3072	0.25	296	21

	Normalized Values					
Healthy	10	10	10	10	10	10
Mild Form of NK	9	6.5	9	3.75	8	8
Severe Form of NK	2	2	6	0.4	7	3
